# “Being in good hands”: next of kin’s perceptions of continuity of care in patients with heart failure

**DOI:** 10.1186/s12877-019-1390-x

**Published:** 2019-12-26

**Authors:** Malin Östman, Siv Bäck-Pettersson, Ann-Helén Sandvik, Annelie J. Sundler

**Affiliations:** 1 Faculty of Caring Science, Work Life and Social Welfare, University of Borås, SE-501 90 Borås, Sweden; 2 Närhälsan Källstorp Health Centre, Region Västra Götaland, Trollhättan, Sweden; 3 Research and Development Primary Health Care Fyrbodal, Region Västra Götaland, Vänersborg, Sweden

**Keywords:** Continuity of care, Heart failure, Next of kin, Phenomenography, Qualitative research

## Abstract

**Background:**

Heart failure (HF) is a chronic condition with a variety of diverse symptoms. Patients with HF are usually elderly with multimorbidity, which are both multifaceted and challenging. Being a next of kin to patients with HF is described as a complex task consisting of managing care and treatment, monitoring illness and being an emotional support, while also being able to navigate the healthcare system especially in long-term contact. However, few studies have investigated next of kin’s perceptions of continuity of care in connection with HF. The present study aimed to describe continuity of care as perceived by the next of kin who care for patients with HF.

**Methods:**

This study used a qualitative descriptive design. Semi-structured interviews were conducted with the next of kin (*n* = 15) of patients with HF to obtain their perceptions of continuity of care. A phenomenographic analysis method was used to capture the participants’ perceptions of the phenomenon.

**Results:**

The analysis reveals that the next of kin perceive that support from healthcare professionals was strongly associated with experiences of continuity of care. Four categories reveal the next of kin’s perceptions of continuity of care: Want to be involved without being in charge; A desire to be in control without acting as the driving force in the care situation; A need for sustainability without being overlooked; and Focusing on making life meaningful while being preoccupied with caregiving activities.

**Conclusions:**

Next of kin perceive continuity of care, when they have access to care and treatment and when caregivers collaborate, regardless of healthcare is given by primary care, municipalities or specialist clinics. A sense of “being in good hands” sums up the need for continuous support, shared decision-making and seamless transitions between caregivers. It seems important that healthcare organisations safeguard effective and collaborative models. Moreover, professionals need to plan and perform healthcare in collaboration with patients and next of kin.

## Background

Heart failure (HF) affects people worldwide and has a significant impact on public health with increased morbidity and high mortality rate [1]. HF is a chronic condition that involves gradual deterioration of the heart’s capacity, resulting in a variety of symptoms, such as shortness of breath, oedema and fatigue. The prevalence of HF is about 1–2% in developed countries and the risk of HF increases with age [2, 3], and in Sweden the prevalence is 20% for people over the age of 80 [4]. Those living with HF are usually elderly, with multiple diseases, although there are variations based on age and gender [5]. This multimorbidity is multifaceted and challenging because it causes disability that results in the need for extensive care and support [6–8].

Patients with HF have an increasing need for healthcare as the condition gradually deteriorates. In Europe including Sweden, most of these patients are taken care of in primary care settings, but some of them also need intermittent specialist care in HF clinics. Frequent hospital admissions and palliative care might be needed as the disease progresses [9]. HF patients require long-term care, and healthcare professionals stress that this can be problematic, given high personnel turnover, resulting in both lack of care continuity and care coordination [10]. Since, the course of the disease is fluctuating, patients need support from both next of kin and healthcare professionals [9, 11, 12]. In the present study, the term, next of kin, refers to the person that patients define as their close family member [13]. Next of kin’s support and participation is regarded as a natural part of caring worldwide, but it can also be experienced as a care burden for the person with that designation [14]. A Swedish study found that living as a next of kin of a patient could be similar to “sitting as a co-passenger in a roller coaster without a seat belt” [11].

Being a next of kin has been described as a complex task that requires managing care and treatment, monitoring illness and being emotionally supportive, while also being able to navigate the healthcare system [15]. They are unprepared for the caring role, which is described as both overwhelming and emotionally stressful; their daily lives are affected and some experience reduced quality of life [16, 17].

Continuity of care is regarded to be particularly important for patients with HF and their next of kin [7, 18]. Haggerty et al. [19] defined continuity of care as the degree to which healthcare events are experienced as coherent, connected and consistent with the patient’s medical needs and personal context. This definition emphasises the patient’s perspective including personal relationships, communication and cooperation. In a review of the concept continuity, Uijen et al. [20] show that the definition of the concept has changed over time and are related to several other concepts, for instance integrated care. In USA, integrated care is used interchangeably with managed care. In the UK with shared care. In other European countries it is similar to seamless care, continuous care and multidisciplinary care. Integrated care includes communication and cooperation between various caregivers with the purpose to merge the patient’s separate care events to a whole, in order to achieve maximum level of continuous care [20, 21]. Integrated care seems to be based on an organisational perspective and doesn’t include personal relationships [20]. In this article the definition of Haggerty et al. [19] was used.

Some studies have emphasised that patients experience continuity of care when they are given information, feel confident and secure about the care pathway and have a relationship with a trusted clinician [22, 23]. Next of kin have defined continuity of care as the need for information, accessibility and continuous contact with different healthcare providers [24]. Others have experienced great demands when caring for a patient with HF, and their need for support is ignored by healthcare professionals [14]. Bodenheimer [25] stated that fragmented care, conflicting advice and inadequate coordination of care and treatment contributes to lack of continuity of care. He suggests the need of new collaboration models that include healthcare professionals, patients and their families to reduce fragmentation and enhance continuity of care. A Swedish study showed that there is a great potential for improvement regarding access to care and treatment over time in patients with HF [26].

Patients’ experiences of continuity of care are well studied [20–22], but next of kin’s perceptions of continuity of care seems to be less studied in the context of HF. Therefore, the present study aimed to describe continuity of care as perceived by the next of kin who care for patients with HF.

## Methods

### Design

This qualitative descriptive study was based on a phenomenographic approach. Phenomenography aims to describe and systematise the variations in people’s lived experiences and their perceptions of a certain phenomenon in the world, given that there is only a limited number of ways in which a phenomenon is perceived [27]. The method allows for descriptions of people’s perceptions in a given situation [28]. The result of this type of analysis is presented qualitatively as different descriptions of categories based on the participants’ perceptions of the studied phenomenon [29].

### Participants and recruitment procedure

The participants (*n* = 15) were recruited from four healthcare centres and two HF clinics in hospitals in the western part of Sweden. A purposeful sample of next of kin was invited to participate in the study. The inclusion criteria were adult next of kin over 18 years, who wanted to participate and were able to communicate their experiences. The patients themselves defined who they considered as next of kin. To obtain varying experiences of the phenomenon of continuity of care, variations was sought regarding gender, age, occupation, the relationship with the patients as well as the number of care contacts the last year. Ten women and five men, ranging in age between 33 and 82, with an average age of 68 years, participated in the study. The participant’s characteristics are presented in Table 1.

**Table 1:** Characteristics of the study participants (*n* = 15)

Gender	Female	*n* = 10
	Male	*n* = 5
Age	Range Mean	33–82 68
Relationship	Wife	*n* = 7
	Husband	*n* = 3
	Living apart	*n* = 1
	Daughter	*n* = 2
	Son	*n* = 2
Occupation	Working	*n* = 3
	On sick-leave	*n* = 1
	Retired	*n* = 11
Duration of heart failure	< 1 year	*n* = 2
	> 1–5 year	*n* = 6
	> 5 year	*n* = 7
Care contacts during the last year	1–2 times	*n* = 3
	3–4 times	*n* = 1
	5–6 times	*n* = 2
	> 6 times	*n* = 9

Information about the study was sent by mail to primary healthcare managers and to the manager of the HF clinics in the hospital. The request for participants was approved, and designated contact nurses in each unit were informed, in writing and verbally, about the purpose of the study. They provided potential participants with the name, address and telephone number of the first author (MÖ), who sent written information about the study. After one week, potential participants were contacted by telephone, and they were given the opportunity to ask questions about the study. Two of them declined to participate in the study due to a lack of time and energy, the others were scheduled for an interview.

### Data collection

Data were collected through individual semi-structured interviews conducted by the first author (MÖ) from February to December 2018. The interviews, which lasted between 30 and 75 min, were conducted in the participants’ home or in different healthcare settings. To capture a rich variety of experiences about the phenomenon, the interviews were carried out as a dialogue. An interview guide, consisting of a few open-ended questions with a focus on continuity of care, was used to encourage the participants to reflect on their experience and talk freely. After the first interview, minor changes were made to the questions. This interview is part of the material. The overall question was: *Can you, please, tell me about your experiences of continuity of care as a next of kin for a patient with HF?* This initial question was followed-up by questions, such as: *What kind of support from healthcare providers do you need as a next of kin? What is important to you in these healthcare contacts?* Subsequently, follow-up questions were formulated based on the participant, situation and context to obtain descriptions that were as detailed and rich as possible. These included: *Can you describe that? Can you tell me more? Can you give an example?*

### Data analysis

The interviews were recorded digitally and transcribed verbatim. The analysis, which included seven steps, was performed according to the process suggested by Dahlgren and Fallsberg [30]. First, the material was read through several times so the researchers could become familiar with the text. In the second step, significant statements were condensed from the participant’s quotes. In step three, the similarities and differences in the perceptions of the phenomenon were compared. In the fourth step, the material was grouped systematically according to the similarities. In the fifth step, a preliminary description of the categories was articulated. In step six, the categories were classified and labelled with an appropriate linguistic expression. In the final step, the characteristics of the qualitatively different descriptions of the categories were compared and contrasted. There was ongoing interaction between the different steps throughout the analytical process. The analysis was mainly carried out by the first author (MÖ) and validated by the second author (SBP). Subsequently, the analysis and description of the categories were shared and discussed with all the authors until consensus was reached. The results were also discussed in a group of PhD students and researchers knowledgeable in the field of phenomenography.

### Trustworthiness

In this study, to determine the trustworthiness, we have used Lincoln and Guba [31]. To ensure credibility, the various steps of the research process was thoroughly described. To make the relationship between empirical data and descriptive categories clear, quotes from the participants were used. To obtain consistency, the interviews were conducted by the first author, with the same interview guide for all interviews. By carefully describing the research process, the transferability of the result to other contexts can be facilitated according to Lincoln and Guba [31].

## Results

Continuity of care, as perceived by next of kin, was related to their caring role. This role was handled in different ways; consequently, the important aspects associated with healthcare contacts for continuity in care varied. Based on the assumptions of what might be beneficial for the patient, the next of kin assigned different meanings to the care contacts. The analysis revealed that the next of kin perceived that support from professionals was strongly associated with their experiences of continuity of care. Four categories and ten subcategories revealed the next of kin’s perceptions of care responsibility and different aspects of continuity of care (Table 2). In this paper, the interview responses associated with these four categories are presented in the text in italics.

**Table 2:** An overview of the categories and subcategories

Categories	Subcategories
Want to be involved without being in charge	Having access to healthcare Assigning responsibility to healthcare providers Being reinforced by regular follow-ups Expects that care is coherent
A desire to be in control without acting as the driving force in the care situation	Staying up-to date Acting as the patient’s advocate
A need for sustainability without being overlooked	Well-functioning contacts over time Being in good hands
Focusing on making life meaningful while being preoccupied with caregiving activities	Balancing existential concerns and caregiving activities Staying engaged in life, despite caregiving activities

### Want to be involved without being in charge

The next of kin perceive continuity of care when they are involved on their own terms without being in charge. This means having access to healthcare providers and services, assigning responsibility to healthcare providers, being reinforced by regular follow-ups and having expectations that care is coherent.

### Having access to healthcare

Continuity of care implies having access to healthcare providers and services. To next of kin, it is important to be able to easily get in touch with healthcare providers whenever they need, without being referred to different caregivers. The possibility of contacting the healthcare centre or the HF clinic for a visit or being referred to the emergency department for care, provides next of kin with a sense of security and makes life easier.

*“There must be an agreement for us to come. He has been there three times now and received very good treatment. He has received test results after one hour. Doctors and the pacemaker nurse have come if there has been any problem with the heart or the pacemaker. We have both felt secure with the fact that heart failure care is accessible”. (Interview 7).*

Accessibility when booking and rebooking appointments, and the possibility of influencing the visit times, promotes continuity of care.

*“When we have a visit time; it is no problem. But when entering the emergency department with dad who is old and has problems with the heart, I must be grateful if he quickly gets enrolled and gets laid in a bed, because some patients may be forced to stay in the emergency department all night”. (Interview 11).*

To next of kin, it is important that healthcare is organised and user-friendly. In particular, this becomes important when access to care becomes difficult.

*“Those who suffer from chronic heart failure will not normally get up until 11 in the morning. Therefore, it is not wise that they have to come to the heart failure clinic at eight in the morning for checks and blood tests. It is neither good nursing care nor respect for the patient or the next of kin”. (Interview 5).*

### Assigning responsibility to healthcare providers

Next of kin perceive continuity of care when they can assign responsibility to healthcare providers, without losing the opportunity to influence the different phases of the nursing process. Some next of kin are always engaged in every healthcare encounter, while others accompany the patient to the healthcare facilities, even if they do not participate.

*“It was only the first time I followed him to the nurse, since then I have been sitting outside and waiting. // He can handle himself; I don’t need to participate”. (Interview 10).*

Other next of kin always assign the responsibility to healthcare providers, especially when they feel uncertain about how to handle the situation.

*“I’m not knowledgeable enough. No, it is better that they handle everything anyway! // I usually say that I drive you to the hospital and then the others can take care of the rest. I think it is easier that way. If I say something wrong, she would be completely crazy”. (Interview 12).*

Some perceive their care responsibility as a dilemma. On the one hand, they feel a relief about not having to take responsibility; on the other hand, they feel that it is wrong for them to not take on this responsibility.

*“Healthcare doesn’t expect me to take responsibility; it’s probably more social expectations. There are, after all, unspoken demands on how to be a good relative. // I would like to have some life of my own. But when thinking of that this responsibility can go on for dozens of years more, it will, of course, have exceptional consequences for me as a relative”. (Interview 5).*

### Being reinforced by regular follow-ups

For next of kin, the experience of continuity in healthcare is strongly related to the care process; the participants emphasised the importance of follow-up. They noted that they felt a sense of security when follow-ups occur. They want an updated care plan, and they want planned examinations and sampling to be carried out on time and the results to be presented to them. The fact that healthcare professionals keep their promises seems to be strongly related to their perceptions of continuity of care.

*“We have regular visits to the nurse. It’s a visit where the nurse checks how my husband reacts on the increased drug dosage that he gets. // I think the visits are good because we feel confident that someone closely follow up on blood value, blood pressure and how he feels over time”. (Interview 3).*

Continuous follow-ups are perceived as being essential, and they increase the likelihood of receiving professional treatment and reduce the risk of acute deterioration and unnecessary hospitalisation.

*“I want her to be checked often, not that it takes this long time. I don’t even remember when we were there last time with mom. Maybe one year ago! But they said it is the same situation; there is no change. It’s the same damage, the same place; but still, it’s been a year. We should know more”. (Interview 15).*

### Expects that care is coherent

The next of kin perceive continuity of care when the care process is coordinated, regardless of who the caregiver is. They are demanding professional collaboration with seamless care and treatment based on the patient’s needs. However, they believe that continuity of care presupposes that the caregivers cooperate and that the caregiver that is responsible for coordination is clearly identified.

*“The GP at the health centre regards that there are waterproof shots between the hospital and primary care. The hospital takes care of the heart and the health centre takes care of the rest. But the treatment must be continuous, wherever it takes place”. (Interview 6).*

Feeling confident in this context includes receiving medical treatment without interruptions. This includes that the prescriptions are issued as promised and prescribed drugs are available at the pharmacy. Unfortunately, according to the next of kin, that is not always the case.

*“When we were at the nurse last time, I pointed out that the medicines are not enough until the next visit. She promised to make sure that a new prescription was printed. But this was never done; there was no prescription. // Now when we do this week’s pill box, then we must take from these pills with a lower dose. We will be able to do it this week but then there is no medicine left”. (Interview 2).*

Continuing medical treatment also consists of pacemaker therapy, an essential part of HF treatment. This includes having access to the best possible equipment. It also means that healthcare professionals can regulate the pacemaker based on the patient’s condition.

*“The pacemaker has functioned without interruption, but we do have the best pacemaker, a “Rolls Royce” with a defibrillator”. (Interview 9).*

### A desire to be in control without acting as the driving force in the care situation

Next of kin perceive continuity of care when they feel involved and have control over what is happening in the care situation without being the driving force. By staying up-to-date and sometimes acting as the patient’s advocate, they strive to facilitate the patient’s complex illness situation.

### Staying up-to date

Staying up-to date makes it easier for next of kin to manage the illness situation. To preserve continuity of care, next of kin use the gathered information from healthcare visits, the internet and brochures or friends. Some of them actively exchange information during the visits, partly to support the patient in the communication process and partly to obtain answers to their own questions and concerns.

*“I ask quite a lot and I don’t think that they experienced me as bothersome when I ask. I want facts, because when we get home, he doesn’t remember and then I have to be able to tell what they said. He can’t always take it; it differs from time to time, but of course he trusts that we are four ears”. (Interview 7).*

Next of kin gain a deeper understanding of the potential problems and how to deal with them by staying up-to date. The more skilled they are at observing and supporting the patient, the easier they can face their own concerns and the uncertainty caused by the illness.

*“The doctor and the nurse don’t really have the same idea about diuretic treatment. The nurse doesn’t want him to use it, so she removes them and the doctor adds them. But I don’t give a shit about it! He still gets a whole tablet of diuretic, because he coughs much more when we try to reduce to half a tablet”. (Interview 2).*

### Acting as the patient’s advocate

Next of kin perceive continuity of care when acting as the patient’s advocate. To mediate the patient’s needs, and understand and manage what is said during the visits, they must take the initiative during the encounters. This is particularly essential when the information between healthcare providers is not coordinated.

*“There are no follow-ups from the hospital, but sometimes they say that you should contact your health centre. But when you do that, they don’t receive any papers or journal about him. Then you have to go there and tell everything over and over again. That’s tough. You have to be firm as a relative to speak for a patient who cannot speak for himself”. (Interview 6).*

Several of the participants highlighted the importance of acting as a link between the patient and the care the patient receives. This is especially important when the patient neither understands the language nor feels at home in the Swedish culture. In these situations, the next of kin acts as an interpreter and tries to mediate the patient’s needs and wishes, linguistically.

*“Mom and dad don’t feel at home in Sweden; they feel outside the healthcare system. It is a difference if I interpret or if they are alone with an authorised interpreter. It’s not just about informing or understanding the language; the authorised interpreter doesn’t know my parent’s situation at all, and everything becomes difficult”. (Interview 15).*

The next of kin stated that they feel obligated when acting as the patient’s advocate, whether they want to or not. While they would like to get away from their care responsibilities, it often works the other way around. Some of them perceive that they are compelled to act and take responsibility for the care when the patient is unable to act accordingly.

“*I do not want to feel that I am responsible for the entire care, being a secretary for the care or act as some kind of assistant nurse who has control so that all medications are correctly handle; but for me, it has actually become like that”. (Interview 2).*

### A need for sustainability without being overlooked

According to the next of kin’s perceptions, continuity of care also means having long-term and well-functioning contacts with healthcare providers over time without being overlooked. In this context, a feeling of being in good hands emerges from the next of kin’s descriptions.

### Well-functioning professional healthcare contacts over time

Well-functioning professional healthcare contacts over time includes encountering nurses or general practitioners (GP) who know the patient’s history, who can reconnect to the previous visits and who care about the patient, the next of kin and their situation. Encounters with openness, understanding and respect create a mutual and trustworthy relationship between the patient, the next of kin and the healthcare professionals.

*“The heart failure clinic means a lot! It feels like we have been connected much longer than a few years. The heart failure nurse is a wonderful person; it feels like she cares a lot about us. She remembers us from time to time and what we have around us”. (Interview 6).*

Some next of kin expressed concern that well-functioning relations would end, while others did not, because they are convinced of being cared for regardless of whom they encounter. For next of kin, both personal chemistry and a personal relationship are required to maintain continuity of care with the patient’s healthcare contacts.

*“The importance of encountering the same person depends on how that person is! Some have been good, others have not. Some persons we didn’t like, but there isn’t much you can do. You have to be patient, but I demand that they take their job seriously”. (Interview 4).*

The interviewees emphasised the importance of getting to know healthcare professionals to establish well-functioning healthcare contacts over time. Next of kin experience continuity of care when they understand the staff’s actions, obtain relevant information and realise that the staff has the patient’s best interests in mind.

*“You know it’s a permanent staff, a nurse who always works with this, who has skills. It is of tremendous importance, that you can trust them, read them and see that they can do their thing”. (Interview 2).*

### Being in good hands

Continuity of care is perceived when next of kin have a feeling of being in good hands, wherever the visits take place. The interviewees perceived that being recognised and confirmed by the healthcare staff in a personal way is very important. This feeling is reinforced by the fact that the healthcare staff greets them and takes time to talk with them.

*“It means a lot that they know who we are, that they recognise us and that they greet us”. (Interview 1).*

External factors, such as knowing where the care settings are located geographically, are important for both planned and emergency visits. It is also important that, during the visit, the atmosphere is permeated by positive attitudes.

*“It was absolutely incredible at the hospital; he was really taken care of there. // It was so nice, and so clean, and so kind staff, all the way down to the cleaner. They came and asked if he wanted something to eat and drink. They were so kind; he could have stayed there for a week.” (Interview 10).*

### Focusing on making life meaningful while being preoccupied with caregiving activities

When next of kin are able to focus on making life meaningful despite their role as a caregiver, they perceive continuity of care. This is also evident when they are able to balance their existential concerns with regard to the patient’s health and well-being, as well as going on living despite their caregiving activities.

### Balancing existential concerns and caregiving activities

Next of kin believe that continuity of care affects their experience of life continuity, and they have to balance existential concerns and care. Although they encounter existential problems, they are trying to preserve continuity in life by addressing concerns, engaging in short-term planning and being present in the moment. Being close to a patient with HF entails caring for that person until the end of life; this can create worries about how to get professional help when the patient’s condition deteriorates.

*“So far, my husband can be alone for a few days; but when he can’t be that anymore, well, then you have to talk to the municipality or something to get relief. That’s when I think if and not when, but I think if so, then we have to solve it then”. (Interview 3).*

Existential problems that arise from the illness situation must be dealt with until the end of life. As the disease progresses, the next of kin must face the fact that the patient changes as a person and that this affects the relationship, both emotionally and practically.

*“I probably haven’t realised how sick she is. In the past, she was very energetic. She arranged everything and initiated happenings. She was the one who initiated spex and excursions. She was impelling, but now it’s nothing. She is busy by herself and her illness”. (Interview 8).*

### Staying engaged in life, despite caregiving activities

The next of kin perceive that their social life is increasingly limited as the patient’s health condition deteriorates. In order to achieve balance in life and go on living, they try to preserve activities of daily living by integrating care contacts into their life planning.

*“We have the calendar, where everything has to be noted regarding the healthcare visits and other things, because we have a little life outside of the healthcare system. Well, the holiday we had been thinking about this spring we must put aside; maybe we can take a last-minute trip, if we get a week free from healthcare”. (Interview 2).*

Going on living despite caring for someone with an illness is possible when next of kin are supported by family and friends. Combining care activities and everyday life may entail getting help with practical tasks and following the patient to healthcare visits. The interviewees noted that it was important to have someone to talk to in confidence about their life situation.

*“My girlfriend has been through the same thing as I before she became a widow. She understands very well what this is all about and we talk a lot about our feelings”. (Interview 7).*

In order to support and keep the family together, next of kin takes responsibility for the patient’s everyday life. This means never leaving the patient alone, not even during a hospital stay.

*“My dad was hospitalised two months ago. I and my family tried to sneak, hide and sit in the corridor, not to disturb the staff. But they were angry with us because we were there; but I cannot leave my parents alone at the hospital”. (Interview 15).*

## Discussion

The study results describe the perceptions that next of kin have about continuity of care as a dynamic process that changes over time, depending on the life situation and the variations in the patients’ health condition.

The interviewees described that their caring efforts interfere with their daily lives, which sometimes is experienced as burdensome. These findings are similar to the results reported in other studies [32–34], which describe how caregiver burden had a negative impact on the partners of patients with HF. However, these experiences can vary depending on the age, gender and health of the next of kin as well as the patient’s need for care [35–37]. At the same time, caring for a patient with HF may have a positive impact on the relationship, creating a sense of closeness and increased social support [38, 39]. According to the next of kin in the present study, continuity of care entails receiving help and support from healthcare providers so they can handle being a carer.

The study’s findings illustrate that next of kin have different perceptions of their role in relation to the healthcare contacts that are needed for a patient with HF. They struggle to manage this in different ways, and their involvement in healthcare processes differ depending on their needs and wishes. This finding is in line with the continuity described by Haggerty et al. [19] in terms of a multidimensional model that highlights the importance of management, information and relational continuity in healthcare. In the present study, continuity of care was emphasised as being linked to the patient’s and the next of kin’s everyday life. Even when trying to cope with illness, a meaningful life is the goal for both patients and next of kin. This put demands on the caregivers to deliver a more person-centred care and that everyone is allowed to be active in the shared decision-making process of care [40]. The next of kin perceived the need to integrate care with everyday life as an existential dimension of continuity of care. This can be understood as the continuity of personal agency, which is a type of continuity that enables people to retain control over their lives in order to manage their health and well-being [41]. Thus, continuity of care seems to be connected to personal agency as an additional dimension of continuity of care.

According to the findings, follow-ups were very important for perceiving continuity of care, regardless of accessibility and care needs. Both accessibility and regular follow-ups of care are regarded as important components to deliver evidence-based care to patient with HF according to European and American guidelines [2, 3]. The importance of follow-ups when living with health problems, especially for patients with HF, has previously been described [42, 43]. A recently published study [18] highlighted the importance of follow-ups in primary care after discharge from hospital. The present study showed how lack of access to follow-ups caused anxiety and uncertainty for the patients and their next of kin because they did not know who to contact if the patient’s condition deteriorated. In the present study, the next of kin seemed to have similar experiences regarding the lack of follow-ups; they expressed worries about how they could get in contact with different healthcare providers. Thus, perceptions of continuity of care are associated with access to healthcare, when needed.

The next of kin expressed various degrees of willingness to participate in the HF patient’s care. They wanted an invitation to participate, to receive information and to be included in the care and treatment. When this happened, they felt more involved, more satisfied and more confident and integrated in care decisions. This is in line with the shared decisions-making process where healthcare professional, patient and their next of kin together make decisions of care and treatment as described by Fitzsimons et al. [44]. This finding is similar to the results reported in other studies [33, 45], which also noted the opposite experiences of being taken for granted, not being informed or being excluded in the care of patients with HF. However, some of the participants in the present study expressed how they were forced to take greater responsibility than requested, by being “the spider in the network”. Instead of perceiving alleviation of care burden, they felt overloaded by different care contacts. In particular, the absence of seamless care, structured follow-ups, information and support from respective caregivers, was regarded frustrating. This can be seen as the opposite to evidence-based HF care [2]. For some next of kin, continuity of care means having control over the patients care process without being forced to act as coordinator in relation to involved caregivers.

The experiences of continuity of care in terms of encountering the same GP is important and well-studied [22]. The present study found comparable results about well-functioning care contacts over time, except that the interviewees’ perceptions of continuity of care also included encounters with nurses, other staff members and the environment, not just GPs. Continuity of care is usually interpreted as a positive phenomenon that contributes to establishing trusting relationships and satisfaction with care from a patient’s perspective, even though it varies [42, 46]. This is similar to the perceptions of continuity of care noted by the next of kin in this study. However, the interviewees in this study pointed out that the perception of relational continuity also depends on the attitudes and behaviour of the caregiver. Thus, continuity of care, in terms of encountering the same caregiver over time, does not help if a person-centred agenda is not in place that takes into account the different needs and resources required to manage everyday life.

A current concept analysis [47], with the intention of clarifying the concept of continuity of care in connection to hospitalisation and discharge, showed that the patient-provider relationship is required for communication and informational continuity, which, in turn, is needed to achieve management continuity with coordinated care over time and in different healthcare settings. However, these findings are not applicable to the present study’s results because the next of kin for HF patients perceived that management continuity, in the sense of having access to various aspects of healthcare, is the basic prerequisite of continuity of care, rather than relational continuity. Perhaps, the necessity of continuity of care varies depending on the context and the next of kin’s needs.

The next of kin for patients with HF expressed various needs for coordinated care in order to overcome obstacles and handle the complex life situation that they encounter following an illness or disease. They want to be met with respect; they also want healthcare professionals to consider the resources they need. This is in line with a person-centred approach that emphasises the importance of coordinated and supportive care for patients with multimorbidity and their relatives [48]. One of the goals of person-centred care is to help a patient create a meaningful life [40]. According to the results of this present study, continuity of care enables the next of kin to focus on having a meaningful life rather than having to ensure that the patient receives the healthcare they need. This improves the quality of the daily lives of both the patient and their next of kin.

### Strengths and limitations

In this study, considerations of trustworthiness were guided by Lincoln and Guba [31]. The core questions in a phenomenographic study are the description of the research process and the relationship between empirical data and categories [49]. To strengthen the trustworthiness and enable the transparency of the analysis, the descriptive categories were illustrated using quotations from the interviews with next of kin. Through direct quotes, the reader has the opportunity to judge the interpretation as well as its relevance to similar contexts. To ensure the credibility of the study, the analysis process was described step-by-step, and the description categories were discussed thoroughly between the authors before consensus was reached [49, 50]. Moreover, trustworthiness was supported with guidance from a research seminar where PhD students and researchers reviewed and discussed the proposed categories and their conformity [50].

Marton [51] stresses that it may be difficult for another researcher to replicate the categories i.e. establish dependability, since phenomenography is about revealing perceptions of unique human experiences. In order to ensure conformability of the process, the first author’s background, preunderstanding and assumptions were considered and discussed. The results of this study cannot be generalised without hesitation. Still, the perceptions of continuity of care can be transferred to other next of kin to chronically ill patients and to similar care contexts.

One limitation of the study may be that the request for participation was made by contact persons, which may have affected who was invited. However, since the contact persons were knowledgeable about the inclusion criteria, this should not have influenced the sampling process. Another limitation may be the issue that continuity of care is an abstract phenomenon. Sometimes, it was easier for the participants to provide examples of what they perceived as non-continuity. This was taken into consideration during the interview where the description of varying perceptions was the focus, and not what was a right or wrong opinion [49].

To ensure variations in how the phenomenon of care continuity was perceived, understood and conceptualised by next of kin (*n* = 15), the data collection was terminated when data saturation was achieved [50].

## Conclusion

Next of kin perceive continuity of care, when they have access to care and treatment and when caregivers collaborate, regardless of healthcare is given by primary care, municipalities or specialist clinics. A sense of “being in good hands” sums up the need for continuous support, shared decision-making and seamless transitions between caregivers. Continuity of care seems to deal with information exchange including that the knowledge of the patient is known to healthcare professionals and that prescribed care measures are performed out. The importance of caregivers’ cooperation to promote continuity seems to be a significant part of the care process in HF. It seems important that healthcare organisations safeguard effective and collaborative models. Moreover, professionals need to plan and perform healthcare in collaboration with patients and next of kin.

## Data Availability

The datasets generated and analysed during the present study are not publicly available because they contain information that could compromise the research participants’ privacy/consent. However, they are available from the corresponding author based on reasonable request.

## Abbreviations

GP: General Practitioner
HF: Heart Failure
